# Sensitivity Enhancement in Magnetic Sensors Based on Ferroelectric-Bimorphs and Multiferroic Composites

**DOI:** 10.3390/s16020262

**Published:** 2016-02-20

**Authors:** Gollapudi Sreenivasulu, Peng Qu, Vladimir Petrov, Hongwei Qu, Gopalan Srinivasan

**Affiliations:** 1 Physics Department, Oakland University, Rochester, MI 48309, USA; sreeni@vt.edu; 2 Electrical and Computer Engineering Department, Oakland University, Rochester, MI 48309, USA; pqu@oakland.edu (P.Q.); qu2@oakland.edu (H.Q.); 3 Institute for Information Systems, Novgorod State University, Veiky Novgorod 173003, Russia; vladimir.petrov@novsu.ru

**Keywords:** magnetic sensor, piezoelectric, ferroelectric, multiferroic, bimorph, bending resonance, proof mass, permanent magnet

## Abstract

Multiferroic composites with ferromagnetic and ferroelectric phases have been studied in recent years for use as sensors of AC and DC magnetic fields. Their operation is based on magneto-electric (ME) coupling between the electric and magnetic subsystems and is mediated by mechanical strain. Such sensors for AC magnetic fields require a bias magnetic field to achieve pT-sensitivity. Novel magnetic sensors with a permanent magnet proof mass, either on a ferroelectric bimorph or a ferromagnetic-ferroelectric composite, are discussed. In both types, the interaction between the applied AC magnetic field and remnant magnetization of the magnet results in a mechanical strain and a voltage response in the ferroelectric. Our studies have been performed on sensors with a Nd-Fe-B permanent magnet proof mass on (i) a bimorph of oppositely-poled lead zirconate titanate (PZT) platelets and (ii) a layered multiferroic composite of PZT-Metglas-Ni. The sensors have been characterized in terms of sensitivity and equivalent magnetic noise N. Noise N in both type of sensors is on the order of 200 pT/√Hz at 1 Hz, a factor of 10 improvement compared to multiferroic sensors without a proof mass. When the AC magnetic field is applied at the bending resonance for the bimorph, the measured N ≈ 700 pT/√Hz. We discuss models based on magneto-electro-mechanical coupling at low frequency and bending resonance in the sensors and theoretical estimates of ME voltage coefficients are in very good agreement with the data.

## 1. Introduction

Ferromagnetic-ferroelectric composites have attracted interests in recent years for studies on the nature of magneto-electric interactions (ME) and for use as sensors, memory devices, and for signal processing [1,2,3,4,5,6]. The coupling between the two subsystems is mediated by mechanical strain. An applied AC field *H* produces a magnetostrictive strain in the ferromagnetic layer, leading to a voltage response *V* in the ferroelectric layer. The ME voltage coefficient (MEVC) = V/(t·H) is a measure of the strength of ME coupling, where *t* is the thickness of the ferroelectric layer. Multiferroic composites studied so far include ferrites, manganites, or transition metals/alloys for the ferromagnetic phase and barium titanate, PZT, or PMN-PT for the ferroelectric phase [6]. A giant low-frequency DME effect was observed in several layered composites [1,2,3,4,5,6,7,8,9,10]. A related ME phenomenon of fundamental interests is the coupling at bending resonance or electromechanical resonance (EMR) modes in the composite [6]. When the AC field is tuned to these modes, MEVC increases by orders of magnitude.

A new generation of magnetic field sensors based on layered composites of ferromagnetic and ferroelectric/piezoelectric phases has been reported in recent years [10,11,12,13,14,15]. Such sensors generally require a bias magnet for operation since strong ME interactions are achieved only when a DC bias *H_b_* is present. In our previous studies we were able to demonstrate the elimination of the need for bias magnetic field with the use of functionally-graded ferromagnetic layer in the composite. In composites of PZT and magnetization (*M*) graded ferromagnetic layer consisting of Ni (with 4 *πM* = 6 kG) and Metglas (4 *πM* = 21 kG) a strong ME coupling was measured at zero-bias (*H_b_* = 0) [16,17]. The coupling is due to the interaction of out-of-plane internal magnetic field arising from grading in *M* and the AC magnetic field *H*. Sensors based on these functionally graded multiferroics were shown to have sensitivities somewhat smaller than those sensors operating under a DC bias magnetic field [11,12,13,14,15,16,17]. The multiferroic magnetic sensors are potentially useful for medical imaging and security-related applications [18,19]. At present there are practical difficulties for large scale use of biomedical imaging techniques such as magneto-cardiography due to the need for expensive superconducting quantum interference device (SQUID) sensors that also have the size disadvantage. There is critical need for passive, room-temperature sensors such as ME sensors that could replace SQUID sensors.

This report is on novel pT-magnetic sensors in which a permanent magnet proof mass is used and, therefore, eliminating the need for a bias magnetic field [5]. Two different types of sensors are discussed: (i) a sensor based on ME coupling in a PZT bimorph with a permanent magnet proof mass and has the advantage of not requiring a ferromagnetic-ferroelectric composite for operation and (ii) a multiferroic sensor of layered PZT-Ni-Metglas. The PZT-bimorph sensor consisted of epoxy bonded two oppositely poled PZT platelets and NdFeB permanent magnet proof mass. A giant magneto-electric effect with MEVC of ~28 V/cm·Oe at low frequencies and enhancement to ~500 V/cm·Oe at bending resonance have been measured for the sensor. The measured equivalent magnetic noise is on the order of 100 pT/√Hz to 10 nT/√Hz at 1–10 Hz. When the AC magnetic field is applied at the bending resonance for the bimorph the measured equivalent magnetic noise is ≈700 pT/√Hz.

In the case of multiferroic sensor with neodymium (NdFeB) magnet proof mass, the ME coupling arises due to magnetostriction in an AC field and also interaction between applied AC magnetic field and *M* of proof mass. The sensor has been characterized in terms of low frequency and resonance ME effects as a function of the proof mass and noise. It is shown that the use of active proof mass enhances the ME sensitivity at low frequency by an order of magnitude and a corresponding decrease in the magnetic noise. Models have been developed for both types of sensors and are based on equations for the strain and electric displacement of piezoelectric bimorph or ME composite due to interaction between *H* and *M*. For finding the low frequency and resonance ME voltage coefficients, we solve elastostatic and electrostatic equations in PZT, taking into account boundary conditions. The MEVC has been estimated as a function of frequency and is found to be in very good agreement with the data. In the sections to follow we discuss the sensor fabrication and characterization and models for the sensors.

Due to its relatively high sensitivity, the ME sensor discussed in this work can be potentially used in cases where measurement of pT-magnetic field is necessary. For instance, it could be a potential candidate for medical imaging applications such as magneto-cardiography in which magnetic fields involved in cardiology events are sensed and dynamic images are reconstructed for disease diagnosis. Compared with other sensors, the ME sensors in this work feature small size, low cost (compared to SQUID), high sensitivity (compared to Hall sensors), and possible miniaturization if MEMS integration technology is utilized.

## 2. Experiment

**Figure 1:**
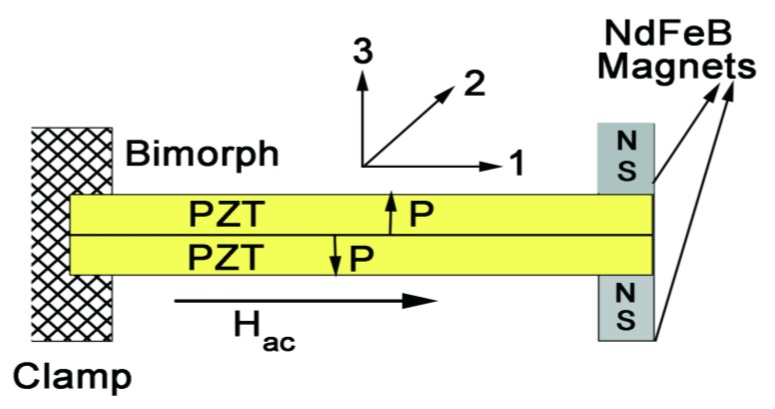
Diagram showing a cantilever of PZT-bimorph with NdFeB permanent magnet proof mass.

The PZT-bimorph sensor studied here is schematically shown in Figure 1 and consisted of a cantilever of two oppositely poled piezoelectric layers of length 50 mm, width 10 mm, and thickness 0.15 mm. We used commercial PZT (#850 obtained from APC International, PA) platelets that were poled by heating to 400 K and cooling to room temperature in a field of 30 kV/cm. The PZT platelets were bonded to each other with a 2 μm thick (West Systems) epoxy. Similarly, for the multiferroic sensors we used 0.16 mm thick Ni foil (99.8% pure and annealed) and 25 μm thick Metglas ribbons (2605SA1, Metglas, Inc., USA) for the ferromagnetic layer and the same samples of PZT as for the bimorph (# 850, APC) were used. Composites of PZT-Metglas-Ni were made by bonding a 6 cm × 1 cm × 0.03 cm PZT to a single layer of Ni and three layers of 25 μm thick Metglas of similar lateral dimensions. The PZT with silver electrodes was initially poled in an electric field and then was bonded to Ni and Metglas with 2 μm thick epoxy layer.

The PZT-bimorph or PZT-Metglas-Ni composite was clamped at one end and a magnet assembly of two NdFeB magnets was epoxy bonded to top and bottom of the bimorph at the free end as shown in Figure 1. The use of NdFeB magnet is due to its high output magnetic flux-to-mass ratio, commercial availability and various shapes for convenient applications. The magnets were discs of diameter 5 mm, 10 mm in height, and mass of 2.5 g each. The remnant magnetization *M* of NdFeB magnet assembly (along direction *3*) was measured to be 15 kG. An AC magnetic field *H* generated by a pair of Helmholtz coils was applied parallel to the sample length (direction *1*) so that interaction with *M* gives rise to a strain in PZT resulting in a voltage *V* across the thickness. In the bimorph, since the PZT platelets are poled in opposite directions and the strain produced is compressive in one of them and tensile in the other, the ME voltage in PZT layers (measured across the thickness along direction *3*) will be of opposite sign so that the overall ME response is enhanced with the use of a bimorph [5].

Measurements of ME sensitivity and magnetic noise were carried out by placing the sample in a plexiglass holder in magnetically shielded μ-metal chamber surrounded by an acoustic shield. The sample clamped at one end was subjected to an AC magnetic field *H* produced by a pair of Helmholtz coils powered by a constant current source (Keithley, model 6221). The ME voltage generated across the thickness of the bimorph was measured with a signal analyzer (Stanford Research Systems, model SR780). Since the ME voltage across PZT is non-uniform along the length of the bimorph, we measured the ME voltage V close to the clamped end where one expects maximum value [20]. The ME sensitivity *S* = *V/H* and the ME voltage coefficient MEVC = *S/t* (*t* is the PZT thickness) were measured as a function of frequency and at room temperature. Measurements of sensor noise were performed with the signal analyzer and was converted to equivalent magnetic noise.

In the case of the multiferroic sensor, measurements of the ME voltage coefficient as a function of frequency and bias magnetic field and equivalent magnetic noise were first carried out without the proof mass and then with the proof mass.

## 3. Results

### 3.1. PZT-Bimorph Sensor

**Figure 2:**
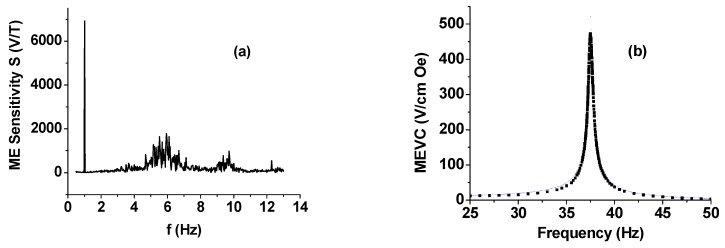
(**a**) ME sensitivity S *vs.* frequency f profile showing the sensor response for H at 1 Hz; (**b**) MEVC *vs.* f data showing resonance enhancement in MEVC at the bending mode for the bimorph-proof mass system.

The ME sensitivity *S* and MEVC were measured by measuring the voltage induced in the bimorph due to the applied AC filed *H*. Two sequential responses of the sensor are examined. First, the ME sensitivity is investigated with a fixed AC field *H* at low frequency far from the structural resonance as an input. Figure 2a shows the representative results on S *vs*. f for the specific case of H = 1 m·Oe at 1 Hz. The ME voltage at 1 Hz measured across the bimorph was V = 680 μV, corresponding to S = 6800 V/T and MEVC = 23 V/cm·Oe. Due to the high sensitivity, the sensor response manifests as a sharp spike in Figure 2a. Figure 2a also shows the noise spectra for frequencies up to 14 Hz., although relatively large background noise levels are seen for frequencies from 5 to 7 Hz and over 9–10 Hz. Second, MEVC *vs*. f for a frequency range of 25–50 Hz is measured, as shown in Figure 2b. This frequency range covers the bending resonance mode of the sensor structure that can be tuned by the attached NdFeB magnet. In the evaluation of a magnetic sensor, MEVC is widely used as a major performance parameter. It is obtained through the sensor response to the applied AC magnetic field amplitude at low frequencies or at resonance. We measured linear V *vs*. H characteristics at all frequencies for all of the sensors discussed in this work. The MEVC = V/(t·H), where t is the thickness of the PZT bimorph, is the estimated form the slope of the ME voltage *versus* H. One observes frequency-independent MEVC, except for the frequency range 33–43 Hz, which shows a resonance enhancement. The MEVC increases rapidly with increasing f from 30 V/cm·Oe at 30 Hz to a peak value of 480 V/cm·Oe at 38 Hz. With a further increase in f, the MEVC decreases rapidly and levels off at ~10 V/cm·Oe for *f* > 47 Hz. The peak in MEVC is associated with the bending resonance in the bimorph with the proof mass [20]. Similar resonances in MEVC are reported for bending modes and longitudinal and thickness acoustic resonance in ferromagnetic-piezoelectric composites [7,8,9,10]. The ME coupling at resonance is due to the traditional strain-mediated coupling, but the AC field is applied at the bending mode frequency so that the overall strain and the strength of ME coupling are enhanced. Data in Figure 2 reveal an increase in MEVC at resonance by a factor of 24 compared to low frequency values. Thus, our PZT bimorph-magnet proof mass system show a large ME effect both at low frequencies and at bending resonance. It is noteworthy here that the permanent magnet proof mass provides an avenue for control of the resonance frequency. The bending mode frequency is found to decrease with increasing proof mass.

Now we compare the results on MEVC in Figure 2 with reported values for similar sensors. Xing, *et al*. investigated the ME coupling in a PZT-bimorph loaded with a permanent magnet tip mass and measured MEVC = 16 V/cmOe and 250 V/cm·Oe at low frequency and bending resonance, respectively [5]. Thus, the MEVC in our case are a factor of two higher than reported values in [5]. Past studies in the case of ferromagnetic-ferroelectric composites include ferrites, lanthanum manganites, 3-d transition metals, and rare earths and alloys for the ferromagnetic phase and PZT, lead magnesium niobate-lead titanate (PMN-PT), quartz and AlN for the ferroelectric/piezoelectric phase [1,2,3,4,5,6,7]. The ME sensitivity at 1 Hz in Figure 2 is two orders of magnitude higher than reported values for bulk ferrite-piezoelectric composites and for bilayers and trilayers of ferrite-PZT and lanthanum manganite-PZT [8]. Additionally, it compares favorably with MEVC of 3–52 V/cm·Oe at 1 kHz for Metglas composites with PZT fibers and single crystal PMN-PT [1]. The resonance MEVC in Figure 2 is higher than for ferrite-based composites, but is smaller than the best value of ~1100 V/cm·Oe reported for Metglas-PMN-PT [1].

**Figure 3:**
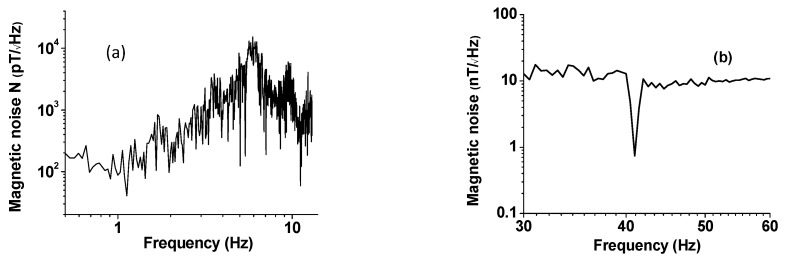
(**a**) Equivalent magnetic noise *N* as a function of frequency for the PZT-bimorph sensor; and (**b**) Results as in (**a**), but for frequencies centered around the bending resonance in the sensor. The minimum in N occurs close to bending mode frequency for the cantilever sensor.

Next we discuss the noise measurements on the bimorph for possible use as magnetic sensors. Data on equivalent magnetic noise floor were obtained over 0.5–50 Hz. The equivalent magnetic noise *N* in terms of *T/*√*Hz* was estimated from the measured noise (in V/√Hz) and the ME sensitivity *S* (in *V/T*) from data in Figure 2. Results on low-frequency *N* *vs*. *f* are shown in Figure 3a for our samples with PZT bimorph and magnet tip mass. One notices a general increase in *N* from 100 pT/√Hz at 1 Hz to ~1 nT/√Hz at 10 Hz. The data on noise N *vs*. *f* over 30–60 Hz in Figure 3b shows a constant value of *N* = 10 nT/√Hz away from bending resonance frequency and *N* decreases sharply to ~700 pT/√Hz close to the bending mode frequency. Minor peaks of unknown origin are seen above and below the resonance frequency. Now, we compare the *N*-values for our sensor with reported values for ferromagnetic-piezoelectric sensors. The best *N* values reported to date are for PZT or PMN-PT fibers and Metglas-based sensors. Gao, *et al.*, in their work on comparison of sensitivity and noise floosr for ME sensors, reported *N* ranging from 20–150 pT/√Hz (at 10 Hz), respectively, for Metglas with PZT fibers or single crystal PMN-PT [21]. Wang, *et al.* reported a further reduction in *N* to 5 pT/√Hz at 1 Hz for Metglas/PMN-PT fiber sensors [1]. Thus, the magnetic noise for the sensor studied here is much higher than the best reported values for multiferroic composite sensors [22].

### 3.2. Multiferroic Sensor

**Figure 4:**
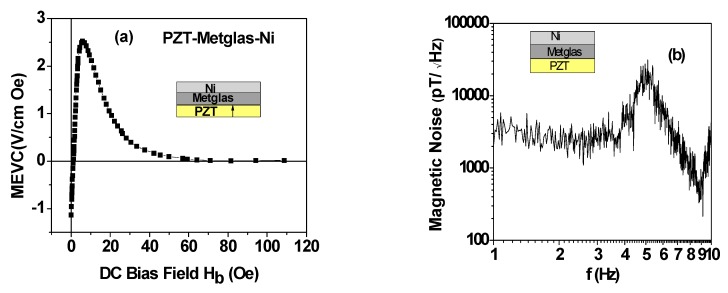
(**a**) ME voltage coefficient as a function for bias magnetic field H_b_ for the multiferroic composite without the proof mass; and (**b**) Equivalent magnetic noise *versus* frequency under H_b_ = 0.

Similar ME voltage coefficient and noise measurements were done on the PZT-Metglas-Ni composite. The specific thickness of 75 μm for Metglas was chosen based on our observation of the maximum ME response for samples with three layers of 25 μm thick ribbons. Measurements of ME coupling strength were done for the following conditions: (i) as a function of bias field and frequency for the composite without the proof mass, and (ii) with the poof mass and as a function of the mass of the NdFeB magnet. Figure 4 shows data on MEVC as a function of bias magnetic field for the AC field applied at a frequency of 30 Hz and magnetic noise N *vs*. f. The bias field *H_b_*-dependence of MEVC is shown in Figure 4a. As *H_b_* is increased from zero one observes a decrease in the magnitude of MEVC from 1.2 V/cm·Oe, zero-crossing at *H_b_* = 2 Oe, and is followed by an increase to a peak MEVC of 2.5 V/cm·Oe. With a further increase in *H_b_*, the MEVC decreases rapidly and attains zero value for bias fields higher than 60 Oe. The observation of zero-bias ME coupling in PZT-Ni-Metglas has been discussed in detail in our earlier works [16,17]. Since both the magnetostriction and piezomagnetic coefficient *q* are vanishingly small for zero-bias, the effect cannot be attributed to piezomagnetic strain-mediated coupling at the interface. The zero-bias ME coupling arises due to a transverse magnetization that originates from grading in magnetization, Ni with 4 πM of 6 kG and Metglas with 4 πM of 21 kG. The torque resulting from transverse magnetization at Ni-Metglas interface and the in-plane ac magnetic field gives rise to a strain that results in an ME coupling for *H_b_* = 0. The A theory was proposed for the effect and calculated MEVC was found to be in agreement with the data [16,17]. We refrain from further discussion of this effect in this work.

Figure 4b shows noise N *versus* frequency measured at zero-bias. The noise at 1 Hz is on the order of 3 nT/√Hz and remains constant for *f* = 1–4 Hz. With further increase in *f*, *N* increases to a peak value of 30 nT/√Hz at 5 Hz and decreases to 0.3 nT√Hz at 9 Hz. Thus, the overall equivalent magnetic noise for PZT-Metglas-Ni sensor operating under zero magnetic bias is somewhat higher than for the PZT-bimorph with a permanent magnet.

**Figure 5:**
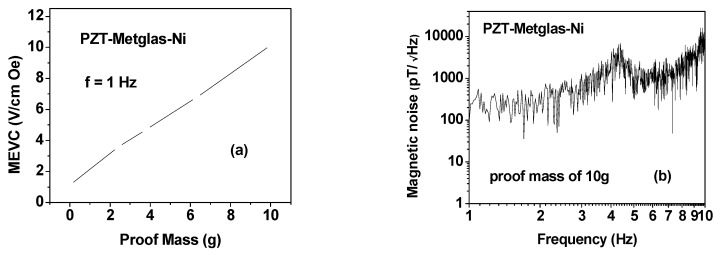
(**a**) Variation of the ME voltage coefficient at 1 Hz with the mass m of the proof mass for the multiferroic composite; and (**b**) *N* *vs*. *f* data for the composite with a proof mass m = 10 g.

Following the noise measurements at zero-bias, we investigated the influence of a permanent magnet proof mass on the ME coupling strengths and noise. Data obtained for low-frequency MEVC and N *vs*. f for this case are shown in Figure 5. The variation in the MEVC at 1 Hz as a function of the mass *m* of each magnet (in the assembly as in Figure 1) is shown in Figure 5a. A four-fold increase in MEVC compared to zero-bias value (in Figure 4) is measured for *m* = 2.5 g. A linear variation in MEVC with m is evident from the data. The noise N *vs*. f data in Figure 5b for *m* = 10 g also shows a substantial reduction in *N* with the use of proof mass compared to the data in Figure 4b. The noise reduction, for example, is by a factor 15 from 3 nT/√Hz to ~200 pT/√Hz at 1 Hz.

**Figure 6:**
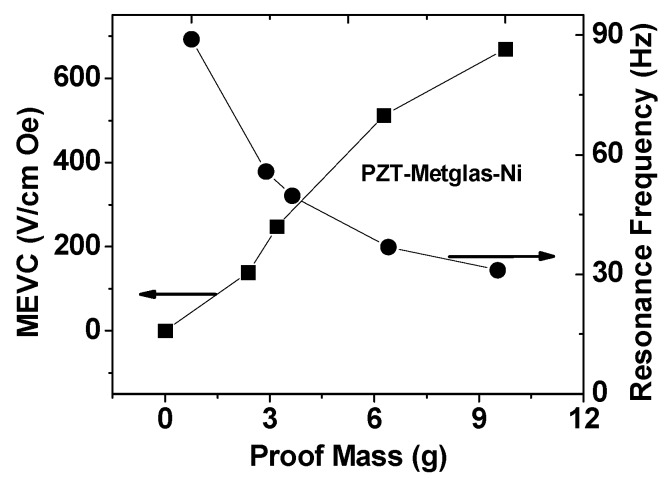
The bending resonance frequency *f_r_* and the MEVC at resonance frequency as a function of the mass m of the proof mass for the multiferroic composite.

Figure 6 shows the peak MEVC at the resonance frequency and the resonance frequency *f_r_* when the PZT-Metglas-Ni is driven to bending resonance with a permanent magnet proof mass m. An increase in MEVC and a decrease in *f_r_* are seen in the data. The increase in MEVC with *m* is approximately linear with a value of 600 V/cm·Oe for *m* = 10 g, but for a given proof mass *m*, the PZT-bimorph has a stronger ME response than for the multiferroic composite.

The low-frequency and resonance MEVC measured for the graded composites with the permanent magnet proof mass are comparable to the best values reported for multiferroic composites. The low-frequency MEVC for the q-graded composites in Figure 5 and Figure 6 are orders of magnitude higher than reported values for bulk ferrite-piezoelectric composites, for bilayers and trilayers of ferrite-PZT, and lanthanum manganite-PZT [8,9]. Gao, *et al*. recently reported on MEVC at 1 kHz for Metglas-based symmetric composites [21,22,23]. Measurements with PZT fibers and single crystal lead magnesium niobate-lead titanate (PMN-PT) or lead zinc niobate-lead titanate (PZN-PT) showed MEVC of 3 V/cm·Oe for Metglas-PZT to 45 V/cm·Oe for samples with PMN-PT [21,23]. The MEVC in our *q*-graded samples in Figure 5a are comparable to reported values for Metglas-PZT-Metglas ([21,23]). Since bending modes are absent in symmetric laminates, we compare the resonance MEVC with that reported for asymmetric laminates. The *q*-graded samples in this study show an order of magnitude higher MEVC than for Metglas/piezoelectric sample with an active tip mass [24].

## 4. Theory and Discussion

A model for the magneto-electric response of the PZT-bimorph and the multiferroic composite with permanent magnet proof mass is considered next. We first discuss the case of PZT-bimorph and then the composite of PZT-Metglass-Ni. The specific focus is on low-frequency ME response and MEVC versus frequency characteristics around the bending resonance frequency. A cantilever with PZT layers in (x,y) or (1,2) plane as in Figure 1 is assumed with one end clamped and the permanent magnet assembly on the free end. The thickness of PZT along *z*-direction (direction 3) is assumed to be small compared to its length or width. The interaction between the AC magnetic field along direction 1 and remnant magnetization of the magnet along direction 3 gives rise to a piezoelectric strain in PZT. Based on equations of bending vibrations [20,25], the general expression for displacement *w* in *z* direction perpendicular to the sample plane can written as:

*u=C_1_ sinh(kx)+C_2_cosh(kx)+C_3_sin(kx)+C_4_cos(kx)* (1)

The wave number *k* is defined by expression:

(2) $$k^{4}=\omega^{2}\frac{2t\rho }{D}$$

where *ω* is circular frequency, *t* is thickness of each layer, *ρ* is density, and *D* is cylindrical stiffness of cantilever. The integration constant in Equation (1) should be determined from boundary conditions that have the following form for the cantilever with an attached permanent magnet at the free end:

(3) w=0 and ∂w∂x=0 for x=0;My=∂w∂x⋅Iω2b+μ0JHvb and Vy=−mwω2b at x=L

where *m*, *v*, *I*, and *J* are mass, volume, moment of inertia of magnet with respect to axis that is positioned in the middle plane along *y* axis, and remanent magnetization, respectively; *M_y_* is the torque moment relative to *y*-axis produced by internal stresses in bilayer per unite width; *V_y_* is the transverse force per unite width; *H* is applied ac magnetic field; and *L* is the sample length.

Induced electric field can be found from the open circuit condition $\int_{G}{}^{1,2}D_{3}dx=0$ where *^1,2^D_3_* is electric induction in first and second piezoelectric layers, *G* is the cross-section of sample normal to the *z*-axis, and ${}^{1,2}D_{3}=\pm d_{31}{}^{1,2}T_{1}+\varepsilon_{33}{}^{1,2}E_{3}$. Here *d*_31_ and *ε*_33_ are piezoelectric coupling coefficient and permittivity of piezoelectric and *^1,2^E* is internal electric field in layers. The stress components *^1,2^T* can be expressed in terms of strain components *^1,2^S* from elasticity equations ${}^{1,2}T_{1}=Y({}^{1,2}S_{1}-d_{31}{}^{1,2}E_{3})$ where Y is the modulus of elasticity of piezoelectric component at constant *E* and ${}^{1,2}S_{1}=-z_{1,2}\frac{\partial^{2}w}{\partial x^{2}}$ (*z_1,2_* is distance of current point of first or second layer from the middle plane). Low frequency MEVC is reduced to the following expression for the 1D case:

(4) $$\alpha_{E31}=\frac{3v\mu_{0}Jd_{31}}{4t^{2}b\varepsilon_{33}}$$

where *t*, *b*, *v*, and *J* are thickness and width of each piezoelectric layer, volume, and remanent magnetization of magnet, correspondingly.

Finally, one can get the following expression for ME voltage coefficient at EMR with the assumption that the moment of inertia of magnet is negligibly small and $K_{31}^{2}<<1$:

(5) αE 31=3vd31μ0J[(r1r3−1)kLmm0+r2r3+r1r4]4bt2kLε33[(r1r4−r2r3)kLmm0+1+r1r3]

where $r_{1}=\cos(kL);\;r_{2}=\sin(kL);\;r_{3}=\text{cosh}(kL);\;r_{4}=\text{sinh}(kL);$

It can be inferred from Equations (4) and (5) that ME coefficient is substantially determined by product of piezoelectric coefficient and remanent magnetization of magnet. Taking into account the magnet mass results in some variation of ME coefficient. EMR frequencies are determined by roots of denominator. The influence of magnet mass on resonance frequencies is specified by the ratio of tip mass to bilayer mass, *m/m_0_* and vanishes when this parameter is much less then unity. A dramatic decrease in EMR frequency occur when the proof mass is of the order of bilayer mass. Expanding the denominator of Equation 5 into series in *kL* to sixth order and solving this equation for *kL* leads to the approximate expression for fundamental EMR frequency:

(6) fr=tπL23Yρ(12mm0+3)

The peak ME voltage coefficient at bending mode frequency can be estimated as:

(7) αE 31r=9Qvμ0Jd31(5mm0+1)40ε33bt2(4mm0+1)

with *Q* denoting quality factor for bending resonance. Taking the total magnetic moment as proportional to magnet mass, expression for peak ME voltage coefficient can be reduced to form:

(8) αE 31r=9Qmμ0σd31(5mm0+1)40ε33bt2(4mm0+1)

where σ is mass magnetization of magnet. Here Q is the quality factor for bending resonance. Resonance losses are taken into account by a using a complex frequency *ω* + *iω*’ with *ω*’*/ω* = *1/*Q, and Q was estimated from observed resonance profiles*.* The following material parameters were used for the calculations: *Y* = 0.65 × 10^11^ N/m^2^, density of PZT *ρ* = 7.7 × 10^3^ kg/m^3^, *d*_31_ = −1750 × 10^−12^ m/V, *ε*_33_/*ε*_0_ = 1750, *m* = 5 g and *μ*_0_*J* = 1.5 T.

**Figure 7:**
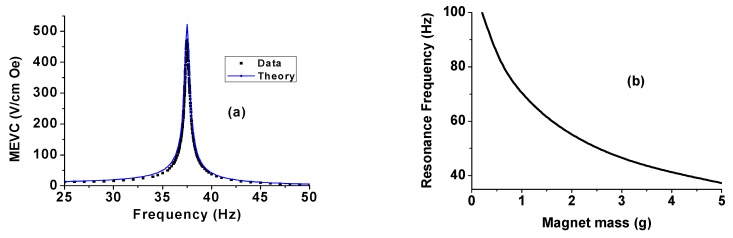
(**a**) Theoretical MEVC as a function of frequency for the PZT bimorph with permanent magnet tip mass. Measured values are also shown for comparison; and (**b**) Calculated bending mode frequency as a function of the mass of permanent magnet.

Theoretical estimates of MEVC *vs.* frequency are shown in Figure 7a. Measured values (in Figure 2) are also shown for comparison. One observes a very good agreement between theoretical MEVC *vs*. f profile and the data. Both the values of MEVC and the bending mode frequency are within 2% of the measured value. Calculated values of the bending mode frequency are plotted as a function of the mass of the permanent magnet in Figure 7b. One infers the following from the results in Figure 7. (i) The cantilever arrangement facilitates electromechanical resonance at low-frequencies compared to longitudinal or thickness acoustic modes; (ii) It is possible to control the resonance frequency with proper choice for the mass of the permanent magnet; and (iii) assuming a linear increase in M with the magnet mass, any decrease in the resonance frequency with increasing m will be accompanied by an increase in the peak MEVC.

Similarly, we derive expression for the MEVC for trilayer of Ni, Metglas, and PZT with active tip mass. A cantilever with Ni, Metglas, and PZT layers in (*x,y*) or (*1*,*2*) plane is assumed with one end clamped and the permanent magnet assembly on the free end. The thickness of the trilayer is assumed to be small compared to its length or width. As opposed to above calculation for PZT bimorph, the expression for the wave numbed takes on the form:

$$k=\sqrt[{4}]{\frac{\omega^{2}({}^{p}\rho {}^{p}t+{}^{m1}\rho {}^{m1}t+{}^{m2}\rho {}^{m2}t)}{D}}$$

The torque moment *M_y_* and transverse force per unite width *V_y_* that enter into the boundary conditions are calculated over the total volume of the trilayer. Finally, assuming the moment of inertia of the magnet to be negligibly small, and electromechanical and magnetomechanical coupling coefficients to be small compared to unity, we obtain expression for MEVC:

(9) αE 31=Ypd31[μ0(pt−2z0)JVbD−(a1m1q11+a2m2q11)][r1r4+r2r3+(r1r3−1)mm0kL]2ε33kL[1+r1r3+(r1r4−r2r3)mm0kL]

with dimensionless factors:

$$\begin{array}{l} a_{1}=\frac{1}{2D}{}^{m1}Y[{\left(z_{0}-{}^{m1}T\right)}^{2}-z_{0}^{2}](t_{p}-2z_{0})\\ a_{2}=\frac{1}{2D}{}^{m2}Y[{\left(z_{0}+{}^{m1}T+{}^{m2}T\right)}^{2}-{\left(z_{0}+{}^{m1}T\right)}^{2}](t_{p}-2z_{0}) \end{array}$$

Assuming the total magnetic moment to be proportional to magnet mass, expression for ME voltage coefficient can be reduced to form:

(10) αE 31=Ypd31[μ0(pt−2z0)σmbD−(a1m1q11+a2m2q11)][r1r4+r2r3+(r1r3−1)mm0kL]2ε33kL[1+r1r3+(r1r4−r2r3)mm0kL]

Equations (9) and (10) show that the contribution of magnetization of the proof mass to ME coupling is similar to that of the piezomagnetic components. The expression enclosed in first square brackets of numerator includes the terms corresponding to the proof mass magnetization and piezomagnetic coupling in two magnetostrictive layers. For zero tip mass, ME response is stipulated by the product of piezoelectric and piezomagnetic coefficients. A non-zero passive load (*J* = 0) results in an increase in the total ME response. This increase is a linear function of *m/m*_0_*.* When employing an active tip mass with *J* ≠ 0, the term quadratic in *m* is added to the product of piezoelectric and piezomagnetic coefficients. In addition, the load mass gives rise to a decrease in EMR frequency.

The approximate expression for fundamental EMR frequency and peak ME voltage coefficient at bending mode frequency can be estimated as:

(11) fr=1πL23Dρ¯t(4mm0+1)

(12) αE 31 r =3QpYpd31(1+5mm0)[μ0(2z0−pt)σmbD+a1m1q11+a2m2q11]20ε33(1+4mm0)

where $\bar{\rho }$ and t are the average density and total thickness of the sample.

The model developed here, therefore, provides several avenues for tailoring the bending resonance frequency and MEVC to achieve desired sensitivity and reduce noise. The sensor of magnetic fields discussed in this report has several unique advantages over traditional sensors such as the Hall effect sensors, SQUID sensors, or magnetoelectric composite sensors. The proposed sensor is passive, does not require a bias magnet for operation, operates at room temperature, and has cost advantages as well. Although temperature certainly will affect the sensor response, it is not the focus of the discussion in this manuscript. Moreover, at this exploratory stage, the external magnetic field is usually generated using a pair of Helmhotz coils, there is no room for a temperature chamber for temperature characterization. Future research will certainly include comprehensive study of sensor responses including the temperature effects.

## 5. Conclusions

Sensors of AC magnetic fields consisting of a PZT bimorph or a multiferroic composite with a permanent magnet for a proof mass have been designed and characterized. The sensor operation is based on magneto-electric interaction mediated by mechanical strain. The applied AC magnetic field interacts with the remanant magnetization of the permanent magnet resulting in a strain that gives rise to a voltage response from the PZT bimorph to PZT in the composite. Magneto-electric characterization of the sensors clamped at one end indicate a giant ME effect both at low-frequencies and at bending resonance and the MEVC are comparable to best values reported for ferromagnetic and ferroelectric composites. The equivalent magnetic noise range from 100 pT/√Hz to 10 nT/√Hz, depending on the frequency. Models for the sensors have been developed and estimates of low frequency and resonance MEVC are in very good agreement with the data. The key advantages of the sensors are (i) the elimination of the need for a DC magnetic bias field that is required for high sensitivity ferromagnetic—ferroelectric magnetic sensors, and (ii) potential for control of the sensitivity by controlling the MEVC and bending resonance frequency with proper choice of proof mass. It is possible to decrease the resonance frequency and increase MEVC by increasing the proof mass so that high sensitivity could be achieved by operating the sensor under frequency modulation [22,26]. Future research will include investigation of effects of other parameters, such as temperature and environmental vibration on the sensors. Suitable technologies, such as MEMS integration for device miniaturization and sensor array implementation, will also be explored.
